# Editorial: Sensor technologies and biosignal processing methods to explore the physiological functions of proprioception

**DOI:** 10.3389/fphys.2023.1172374

**Published:** 2023-03-15

**Authors:** Ahsan H. Khandoker, Ryoichi Nagatomi, János Négyesi

**Affiliations:** ^1^ Healthcare Engineering Innovation Center, Khalifa University, Abu Dhabi, United Arab Emirates; ^2^ Department of Biomedical Engineering, College of Engineering, Khalifa University, Abu Dhabi, United Arab Emirates; ^3^ Division of Biomedical Engineering for Health & Welfare, Tohoku University Graduate School of Biomedical Engineering, Sendai, Miyagi, Japan; ^4^ Department of Medicine and Science in Sports and Exercise, School of Medicine, Tohoku University, Sendai, Japan; ^5^ Department of Kinesiology, Hungarian University of Sports Science, Budapest, Hungary; ^6^ Fit4Race Kft, Budapest, Hungary; ^7^ Department of Medicine and Science in Sports and Exercise, School of Medicine, Tohoku University, Sendai, Japan

**Keywords:** proprioception, motor control, side dominance, balance, gait analyses

Understanding proprioceptive functions in human motor control by applying novel signal processing techniques is essential for sports biomechanics, performance, and kinesiology research. There is even an association between proprioceptive dysfunction and neurological disorders. Researchers with expertise across various disciplines should understand the physiological markers of proprioceptive functions at various levels. This Research Topic aimed to integrate cutting-edge biosignal processing methods from kinesiology, biomechanics, and computational modeling to explore new biomarkers of proprioception.

As Proprioception relates to movement stability and balance, Montull et al. proposed an objective measure of proprioception by showing that assessment of Proprioception and motor training effect (before and after) through wearable accelerometry signals was made possible. They showed that Hurst (H)-exponents of accelerometer signals (from legs and center of mass) estimated by Detrended Fluctuation Analysis were significantly lower in the highly trained group after training.

Unstructured surfaces are widely used in proprioceptive training to improve balance and joint stability. Considering that there have been no studies that have varied the two sides of the Togu Jumper (TJ) and simultaneously monitored leg muscle activation and kinematics around the ankle, knee, and hip joints, Mayer et al. examined leg muscle activity and kinematics in response to a single-leg stance on the two sides of the TJ and the floor. They found greater activation in almost all muscles when balancing on either TJ side compared to the floor. However, no differences were found 1) between the two sides in any muscles or 2) in the equilibrium procedures at the pelvis level.

Ivusza et al. showed that random variation in movement patterns during practice training compared to blocked organization of lower-extremity proprioceptive motor practice did not improve balance and postural adjustments. However, Postural adjustment improved only in the sagittal plane, indicating variations in individual adjustment strategies.

Although corticospinal and spinal level mechanisms may underlie balance control during balance perturbation tasks, the excitatory modulation of responses and voluntary activation is still unclear. Given that the reliability of neither transcranial magnetic stimulation (TMS) nor Hoffmann’s reflex (H-reflex) measurements during high-amplitude balance perturbation tasks is unknown, Hu et al. examined their test-retest reliability as well as corticospinal modulation during such task. They showed that TMS and H-reflex measurements were reliable for different delays between- and within sessions following perturbation, indicating that these methods can be used to measure corticospinal excitability during balance perturbation.

Sun et al. also examined the relationship between posture and H-reflex. However, they compared the neuromodulatory processes of participants with and without peripheral neuropathy (PN) during prone, standing, and the heel-contact phase of walking. Their results indicated that plantar sensory input is important in maintaining standing postural control in the healthy population. The type I afferent fiber reflex loop (H-reflex) contributed more to standing postural control in participants with PN. These results indicate that the H-index parameter may be an excellent method for detecting PN. Still, it is unsuitable for discriminating the severity of PN with impaired foot sole sensitivity.

Because post-stroke individuals have such a high risk of falling during walking with cautious gait adaptations, Nagano et al. investigated if gait retraining through biofeedback technique could reduce their fall risk by changing the strategies in gait control. The study showed that the post-stroke group’s affected limb demonstrated lower mean, higher variability, and lower kurtosis of minimum foot clearance (MFC), the minimum of the foot’s vertical margin from the walking surface. Mid-swing MFC was associated with a risk of falls in the elderly. Post-stroke gait was also characterised by shorter step length, larger step width, and increased double support time. However, future studies should further check if free walking MFC features could be more interesting than that in the treadmill protocols they applied.

Additionally, we (Négyesi et al.) examined the effects of side-dominance on standing stability in healthy young adults using ground reaction force, motion capture (MoCap), and electromyography data, since it appears that central and peripheral functional asymmetry may differ between left- and right-side dominant individuals. This was accomplished by analyzing a multitude of biosignals, including 23 Center of Pressure-related variables, six MoCap variables, and 39 time- and frequency-domain features of Electromyography data from five muscles. It was found that both left- and right-sided participants had a more stable balance when standing unilaterally on their right leg, suggesting that side dominance affects neuromuscular and biomechanical control strategies.

In summary, this Research Topic combines innovative sensing and biosignal processing methods to understand human proprioceptive functions better. However, more collaborative and multidisciplinary efforts should help advance methodological innovations providing great opportunities to increase the clinical applicability of under-explored physiological functions such as proprioception. Future research should focus more on recording physiological data or physio-logging by wearable sensing or Internet of Things (IoT) continuously and daily for better assessment of human neurological activities such as Proprioception. Advancements in data science and processing must complement this multidisciplinary research initiative. Future follow-up research topics in this area should be targeted.

## Abbreviations

H-exponents, Hurst-exponents; TJ, Togu Jumper; TMS, transcranial magnetic stimulation; H-reflex, Hoffmann’s reflex; PN, peripheral neuropathy; MFC, minimum foot clearance; MoCap, motion capture, IoT, Internet of Things.

